# Supplementary material for: Nutraceutical Screening in a Zebrafish Model of Muscular Dystrophy: Gingerol as a Possible Food Aid
Source: Nutrients. 2021 Mar 19;13(3):998. doi: 10.3390/nu13030998 (PMC8003371; doi:10.3390/nu13030998)
Supplement: Supplementary file 1 [file nutrients-13-00998-s001.pdf]

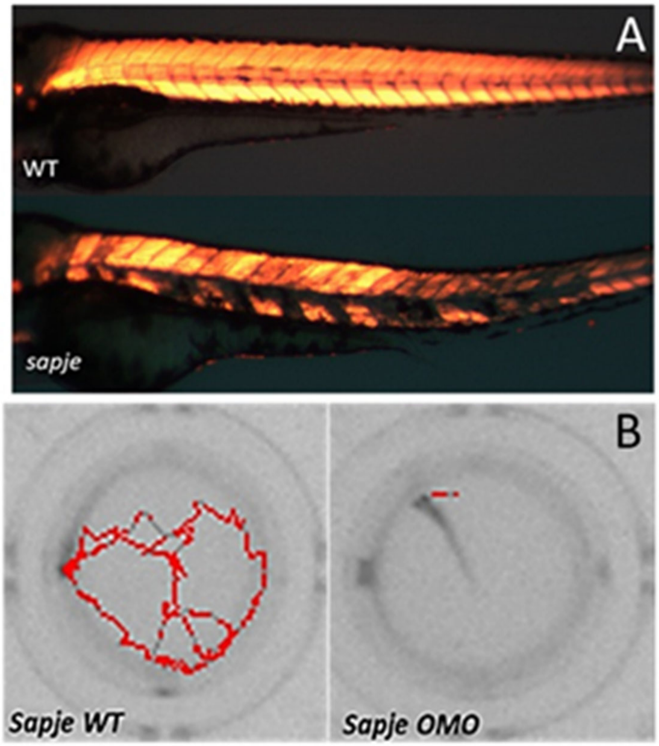

Figure S1. Birefringence assay and locomotion tracking. A) Birefringence assay on wild-type-like non-dystrophic heterozygous (WT) and on dystrophic homozygous *sapje* larvae, at 4 days post-fertilization; B) Example of locomotion tracking of WT and homozygous (OMO) *sapje* larvae.

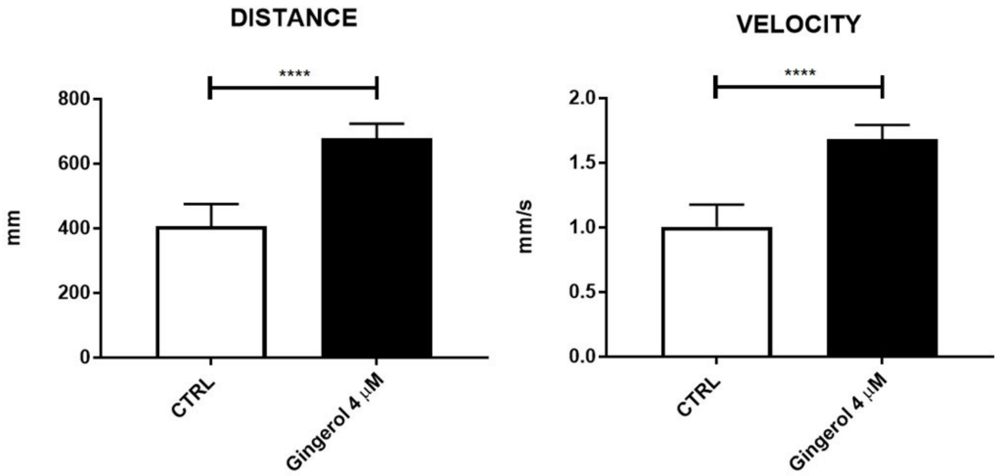

Figure S2. The figure illustrated the effects of Gingerol on locomotion (distance and velocity) of untreated (CTRL n = 103) and treated with Gingerol 4 μM (n = 128), dystrophic homozygous *sapje* larvae at 5 days post-fertilization **** $p \leq 0.0001$.

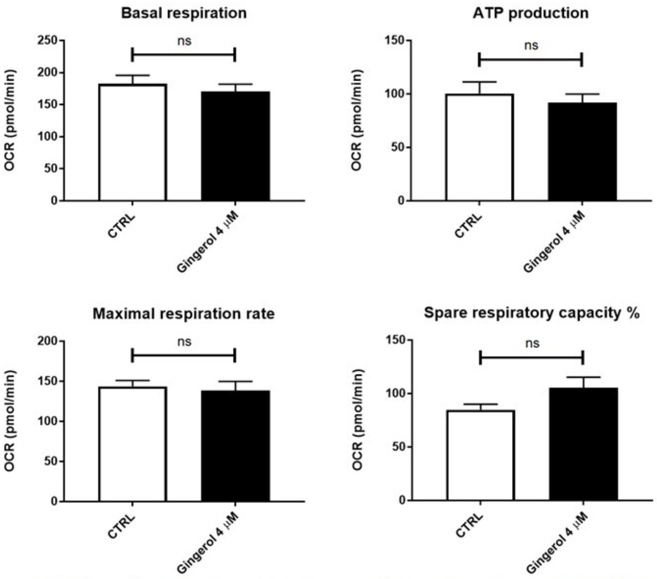

Figure S3. Mitochondrial respiration analysis of untreated (CTRL) and treated with Gingerol 4 μM dystrophic homozygous *sapje* larvae at 5 days post-ferilization, ns $p > 0.05$.
